# Supplementary material for: Dual transcranial electromagnetic stimulation of the precuneus boosts human long-term memory
Source: eLife. 2025 Oct 3;14:RP104220. doi: 10.7554/eLife.104220 (PMC12494378; doi:10.7554/eLife.104220)
Supplement: Supplementary file 1. [file elife-104220-supp1.docx]

Table A.

Experiment 1 statistical details of FNAT and STMB test.

| **Outcome measure** | **Mean (sd)** | | | **Stimulation effect** | |  |
| --- | --- | --- | --- | --- | --- | --- |
|  | **iTBS+sham-tACS** | **sham-iTBS+sham-tACS** | **iTBS+γtACS** | **F_df_** | **p** | |
| **FNAT immediate** | | | | | | |
| TOTAL | 25 (17.9) % [3.0 (2.2)] | 30.8 (21.1) % [3.7 (2.5)] | 40 (21.6) % [4.8 (2.6)] | 7.190 _2,38_ | 0,002 | |
| NAME | 34.6 (21.2) % [4.2 (2.5)] | 38.8 (19.9) % [4.7 (2.4)] | 46.7 (21.2) % [5.6 (2.5)] | 3.200 _2,38_ | 0,052 | |
| OCCUPATION | 50.8 (20.2) % [6.1 (2.4)] | 55.8 (24.9) % [6.7 (3.0)] | 61.3 (19.7) % [7.4 (2.4)] | 2.610 _2, 38_ | 0,086 | |
| **FNAT delayed** | | | | | | |
| TOTAL | 24.2 (19.8) % [2.9 (2.4)] | 26.3 (17.6) % [3.2 (2.1)] | 34.2 (20.4) % [4.1 (2.4)] | 5.860 _2,38_ | 0,006 | |
| NAME | 33.8 (22.7) % [4.1 (2.7)] | 33.8 (19) % [4.1 (2.3)] | 42.9 (21.5) % [5.2 (2.46] | 3.460 _2,38_ | 0,042 | |
| OCCUPATION | 44.2 (22) % [5.3 (2.6)] | 50 (25.5) % [6.0 (3.1)] | 55 (22.7) % [6.6 (2.7)] | 2.880 _2,38_ | 0,068 | |
| **FNAT recognition** | | | | | | |
| TOTAL | 61.7 (24.4) % [7.4 (2.9)] | 65 (20) % [7.8 (2.4)] | 65 (16.6) % [7.8 (2.0)] | 0.401 _2,38_ | 0,673 | |
| NAME | 66.7 (23.4) % [8.0 (2.8)] | 70.8 (16.8) % [8.5 (2.9)] | 71.7 (14.4) % [8.6 (1.7)] | 0.694 _2,38_ | 0,506 | |
| OCCUPATION | 84.6 (15.4) % [10.2 (1.8)] | 88.3 (13.6) % [10.6 (1.6)] | 84.6 (10.9) % [10.2 (1.3)] | 0.733 _2,38_ | 0,487 | |
| **STMBT RT** | | | | | | |
| shape | 1550 (341) ms | 1571 (513) ms | 1597 (503) ms | 0.188 _2,38_ | 0,829 | |
| binding | 1652 (363) ms | 1731 (446) ms | 1809 (454) ms | 2.660 _2,38_ | 0,083 | |
| **STMBT accuracy** | | | | | | |
| shape | 96.5 (4.2) % | 95.8 (4.67) % | 96 (3.12) % | 0.172 _2,38_ | 0,842 | |
| binding | 78 (10.2) % | 78.8 (11.1) % | 77.8 (9.21) % | 0.062 _2,38_ | 0,94 | |

[ ] represent raw scores.
