# Supplementary material for: Dual transcranial electromagnetic stimulation of the precuneus boosts human long-term memory
Source: eLife. 2025 Oct 3;14:RP104220. doi: 10.7554/eLife.104220 (PMC12494378; doi:10.7554/eLife.104220)
Supplement: Supplementary file 2. [file elife-104220-supp2.docx]

Table B.

Experiment 2 statistical details of immediate and recognition FNAT and STMB test.

| **Outcome measure** | **mean (sd)** | | **Stimulation effect** | |  |
| --- | --- | --- | --- | --- | --- |
|  | **iTBS+shamtACS** | **iTBS+γtACS** | **F_df_** | **p** | |
| **FNAT immediate** | 17.5 (8.29) % [2.1 (1.0)] | 26.7 (10.2) % [3.2 (1.2)] | 7.310 _1,9_ | 0,024 | |
| **FNAT recognition** | 43.3 (15.6) % [5.2 (1.9)] | 46.7 (21.9) % [5.6 (2.6)] | 0.255 _1,9_ | 0,625 | |
| **STMBT RT** | | | | |  |
| shape | 1493 (324) | 1547 (389) | 0.348 _1,9_ | 0,570 | |
| binding | 1906 (352) | 1803 (538) | 1.100 _1,9_ | 0,323 | |
| **STMBT accuracy** | | | | |  |
| shape | 95.5 (3.81) | 96.1 (4.25) | 0.310 _1,9_ | 0,591 | |
| binding | 70.6 (12.5) | 76.5 (9.71) | 2.700 _1,9_ | 0,135 | |

[ ] represent raw score
