# Supplementary material for: Dual transcranial electromagnetic stimulation of the precuneus boosts human long-term memory
Source: eLife. 2025 Oct 3;14:RP104220. doi: 10.7554/eLife.104220 (PMC12494378; doi:10.7554/eLife.104220)
Supplement: Supplementary file 3. [file elife-104220-supp3.docx]

Table C.

Experiment 2 statistical details of the delayed FNAT.

|  | **DAY1 mean (sd)** | | **DAY2 mean (sd)** | | **DAY7 mean (sd)** | | **Stimulation effect** | | **Delay effect** | | **Stimulation * Delay effect** | |
| --- | --- | --- | --- | --- | --- | --- | --- | --- | --- | --- | --- | --- |
| **Outcome measure** | **iTBS+sham**  **tACS** | **iTBS+γtACS** | **iTBS+sham**  **tACS** | **iTBS+γtACS** | **iTBS+sham**  **tACS** | **iTBS+γtACS** | **F_df_** | **p** | **F_df_** | **p** | **F_df_** | **p** |
| **FNAT delayed** | 15 (9.46) % [1.8 (1.1)] | 25.8 (10.7) % [3.1 (1.3)] | 14.2 (9.66) % [1.7 (1.2)] | 25 (9.62) % [3.0 (1.2)] | 14.2 (12.5) % [1.7 (1.5)] | 23.3 (12.9) % [2.8 (1.5)] | 8.433 _1,9_ | 0,017 | 0.419 _2,18_ | 0,664 | 0.286 _2,18_ | 7,55 |

[ ] represent raw score
