# Supplementary material for: Dual transcranial electromagnetic stimulation of the precuneus boosts human long-term memory
Source: eLife. 2025 Oct 3;14:RP104220. doi: 10.7554/eLife.104220 (PMC12494378; doi:10.7554/eLife.104220)
Supplement: Supplementary file 4. [file elife-104220-supp4.rtf]

Supplementary File 1 - Table 1. Demographic characteristics.

	Sample (n)	Age (years)*	Sex (female\male)	Education (years)*	
Experiment 1	20	28.6 (4.0)	12\8	18.8 (3.1)	
Experiment 2	10	24.1 (4.7)	6\4	17.4 (4.7)	
Experiment 3	14	27.1 (2.5)	11\3	19.4 (2.1)	
Experiment 4	16	26.9 (2.4)	11\5	18.9 (1.5)	

*Mean (s.d.)
