# Supplementary material for: Dual transcranial electromagnetic stimulation of the precuneus boosts human long-term memory
Source: eLife. 2025 Oct 3;14:RP104220. doi: 10.7554/eLife.104220 (PMC12494378; doi:10.7554/eLife.104220)
Supplement: Supplementary file 5. [file elife-104220-supp5.docx]

Table D.

Subject-specific information about stimulation parameters and e-field calculations.

| **Code** | **Experiment (n)** | **75% eSI (%)** | **Coil-to-cortex distance PC (mm)** | **PC individualized coordinate (x,y,z)** | | | **TMS e-field (V/m)** | **tACS e-field (V/m)** | **TMS+tACS e-field (V/m)** |
| --- | --- | --- | --- | --- | --- | --- | --- | --- | --- |
| 001C | 1,3,4 | 42 | 17.59 | 9 | -31 | 40 | 19 | 0.1 | 19 |
| 002P | 1,3,4 | 46 | 19.75 | -1 | -20 | 56 | 21 | 0.09 | 21 |
| 003M | 1 | 51 | 24.23 | -3 | -67 | 51 | 27 | 0.17 | 25 |
| 004S | 1,3,4 | 44 | 18.62 | 5 | -40 | 50 | 33 | 0.11 | 33 |
| 006L | 1,4 | 43 | 14.61 | 5 | -36 | 56 | 33 | 0.13 | 33 |
| 007S | 1 | 44 | 19.19 | 5 | -32 | 45 | 31 | 0.11 | 31 |
| 008F | 3,4 | 46 | 19.65 | 2 | -47 | 30 | 37 | 0.12 | 36 |
| 009D | 1 | 43 | 18.19 | -6 | -74 | 44 | 25 | 0.2 | 25 |
| 010P | 1 | 39 | 20.09 | -2 | -68 | 52 | 21 | 0.19 | 21 |
| 011V | 1,3,4 | 43 | 20.74 | -1 | -22 | 48 | 30 | 0.11 | 30 |
| 012M | 1 | 44 | 19.82 | 1 | -58 | 63 | 28 | 0.11 | 29 |
| 013D | 1 | 37 | 24.19 | 5 | -75 | 61 | 34 | 0.16 | 34 |
| 014C | 1 | 45 | 19.24 | -3 | -63 | 56 | 32 | 0.13 | 32 |
| 015G | 1 | 44 | 19.53 | 4 | -63 | 57 | 34 | 0.24 | 34 |
| 016L | 1 | 44 | 16.89 | 1 | -66 | 50 | 35 | 0.22 | 35 |
| 017M | 1 | 41 | 19.94 | 10 | -78 | 46 | 28 | 0.14 | 28 |
| 018B | 3,4 | 56 | 24.16 | 0 | -31 | 68 | 22 | 0.11 | 23 |
| 019B | 1 | 45 | 18.99 | 5 | -75 | 41 | 26 | 0.18 | 26 |
| 020D | 1,3,4 | 45 | 16.16 | 7 | -60 | 10 | 38 | 0.24 | 38 |
| 022B | 1,3 | 55 | 20.11 | 2 | -52 | 34 | 31 | 0.17 | 31 |
| 023R | 1,3,4 | 48 | 23.23 | 0 | -49 | 31 | 30 | 0.14 | 29 |
| 024A | 1,3 | 45 | 20.12 | 7 | -41 | 45 | 35 | 0.11 | 35 |
| 035R | 4 | 53 | 18 | -2 | -39 | 39 | 26 | 0.16 | 26 |
| 036M | 4 | 65 | 24.99 | -2 | -30 | 47 | 35 | 0.07 | 35 |
| 037I | 3,4 | 37 | 17.04 | 3 | -37 | 58 | 30 | 0.14 | 30 |
| 038P | 3,4 | 42 | 19.09 | 4 | -31 | 26 | 14 | 0.16 | 14 |
| 039C | 3,4 | 49 | 16.5 | -2 | -36 | 23 | 22 | 0.14 | 22 |
| 040A | 3,4 | 54 | 20.27 | 2 | -50 | 46 | 48 | 0.09 | 48 |
| 041A | 4 | 52 | 21.54 | 0 | -29 | 34 | 25 | 0.09 | 25 |
